# Postoperative opacification of intraocular lens: A case report

**DOI:** 10.1016/j.ijscr.2025.112076

**Published:** 2025-10-16

**Authors:** Qihang Lei, Xiangli Wang, Qin Liu

**Affiliations:** a The First Clinical Medical College of Gansu University of Chinese Medicine, 204 Donggang West Road, Lanzhou, 730000, China; b Gansu Provincial Hospital, 204 Donggang West Road, Lanzhou, 730000, China

**Keywords:** Cataract, Intraocular lens opacification, Hydrogel, Intraocular lens exchange, Case report

## Abstract

**Introduction and importance:**

This case report describes a rare complication of intraocular lens (IOL) opacification in a 76-year-old female patient after age-related cataract surgery in the right eye. Although uncommon, IOL clouding can significantly impair postoperative visual acuity, necessitating surgical intervention. Therefore, choosing the right type of artificial crystal is extremely important.

**Case presentation:**

The patient presented with progressive blurred vision in the right eye for one year. We confirmed the diagnosis and identified the underlying cause after thoroughly excluding other potential influencing factors and make a distinction from congenital cataracts. Subsequently, surgical intervention was performed on the right eye, involving removal of IOL and the re-implantation of new IOL. It resulted in significant improvement in the patient's postoperative vision.

**Clinical discussion:**

Clouding of the IOL following cataract surgery is characterized by a reduction in lens transparency after implantation. Although relatively uncommon, this complication can significantly impair vision. The etiology of IOL opacification post-surgery primarily encompasses material-related factors, surgical factors, patient-specific factors, and other miscellaneous factors. This case underscores the critical importance of evidence-based IOL selection in cataract surgery to mitigate vision-compromising postoperative complications.

**Conclusion:**

With the advancement of surgical techniques and the introduction of new materials, the incidence of IOL opacification following cataract surgery has significantly decreased. Looking ahead, we will prioritize the use of hydrophobic acrylic IOLs or novel anti-calcification IOLs in future surgeries. This strategic choice aims to further minimize the impact of long-term complications on visual acuity post-cataract surgery.

## Highlights

• The article discusses a 76-year-old woman with worsening vision due to a cloudy intraocular lens after cataract surgery, who successfully had it replaced, improving her quality of life and emphasizing the importance of choosing the appropriate type of artificial lens.
• Postoperative opacification of intraocular lenses (IOLs) after cataract surgery can complicate vision recovery, highlighting the need for careful material selection and management, as shown by David J. Apple's lab and Jean Champod's findings.
• IOL opacification is a rare complication of cataract surgery, marked by reduced lens transparency and requiring differentiation from other conditions; hydrophilic IOLs are more prone to this issue, while hydrophobic IOLs offer better stability and lower rates, highlighting the importance of careful IOL selection for better visual outcomes.
• Hydrophilic intraocular lenses can calcify and become opaque, raising postoperative risks; thus, clinicians should consider biological compatibility and material properties when selecting lenses. Looking ahead, we will prioritize the use of hydrophobic acrylic IOLs or novel anti-calcification IOLs in future surgeries.

## 1 Introduction

Post-cataract IOL opacification is a potential complication that may occur following ophthalmic surgery, particularly after the implantation of an IOL during cataract surgery [1]. However, the recognition and study of IOL opacity only began to emerge in the last three decades [2]. Established in 1983 by David J Apple at the University of Utah, the laboratory was specifically designed to investigate the diverse complications associated with IOL implantation. In 1991, Jean Champbell made a groundbreaking discovery: the phenomenon of polymethylmethacrylate (PMMA) IOL opacification. He subsequently sent the removed, opacified IOL to Apple's laboratory for analysis. This pivotal moment marked the world's first documented case of IOL opacification following cataract extraction [3]. As cataract phacoemulsification has become more prevalent and the demand for high-quality postoperative vision has increased, the issue of IOL opacification has garnered significant attention from ophthalmologists both domestically and internationally.

Through the analysis of this case, we have emphasized the significance of material selection and postoperative management in the recovery of patients' vision, providing valuable guidance for future clinical practice.

## 2 Method

The work has been reported in line with the SCARE criteria.

## 3 Case presentation

The patient, a 76-year-old female, presented with complaints of “the vision in the right eye gradually became blurred and it affected my work for one year.” She had no history of hypertension, diabetes mellitus, or trauma. Twelve years prior, she had undergone phacoemulsification with implantation of a hydrophilic acrylate IOL in the right eye. Upon physical examination, the right eye exhibited a visual acuity of 0.1 and an intraocular pressure of 15 mmHg. The IOL showed signs of grayish-white opacification and the contrast and color vision were affected. No other abnormalities were observed in the anterior segment of the eye. Left eye had the same model of hydrophilic IOL with a slight opacities noted. Fundus examination was inconclusive due to the opacity. The social history, family history, drug history, and allergies of the patient were all normal. The patient was admitted with a diagnosis of “IOL opacification following cataract surgery in the right eye.” During her admission, she underwent a comprehensive set of tests, including blood tests for hepatitis B, hepatitis C, syphilis, and HIV, as well as hematologic and biochemical analyses. Additionally, she had chest X-rays performed. All test results were within normal limits, with no significant abnormalities detected.

**Fig. 1:**
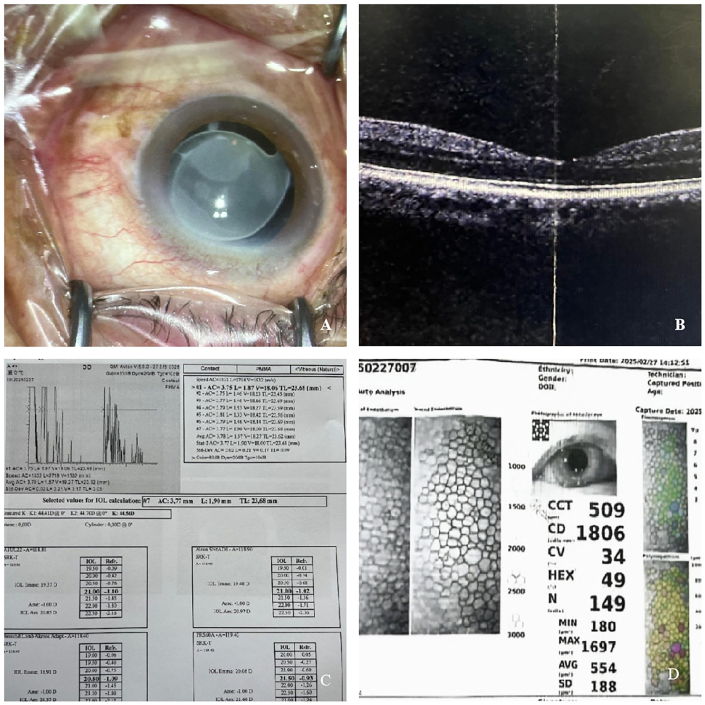
Preoperative photography and examination of the affected segment. A Artificial crystals are grayish-white swollen and cloudy. B No apparent abnormality. C Right eye axis: 23.68 mm. D Corneal Endothelial Count: CD: 1806.

**Fig. 2:**
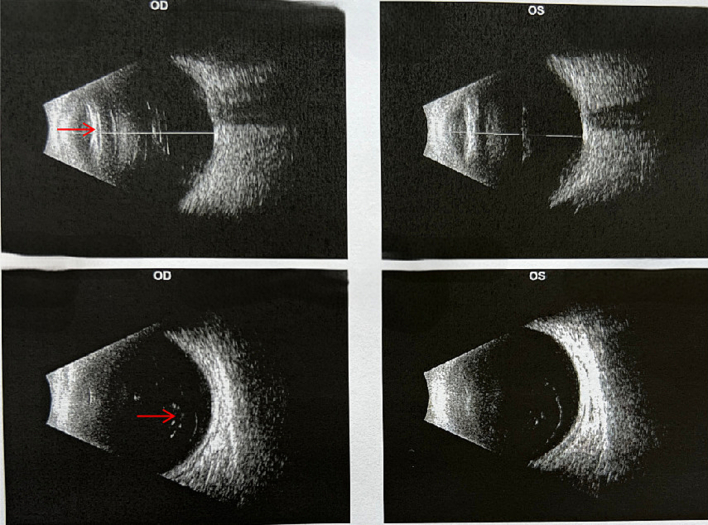
Preoperative ocular ultrasound examination. The crystalline lens of the right eye was cloudy. The vitreous humor of both eyes was cloudy, and no other significant abnormalities were observed.

**Fig. 3:**
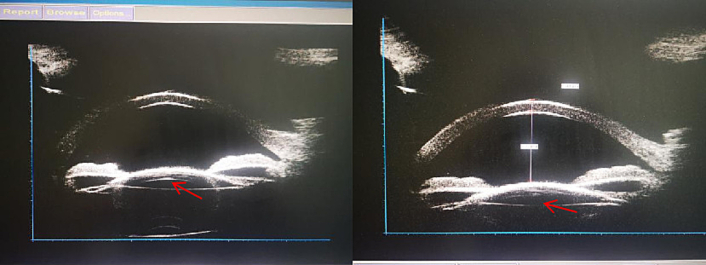
Preoperative ultrasonography of the UBM examination. Photo of the intraocular slit lamp of the invisible intraocular lens.

Upon admission, fundus photography, macular OCT, A-scan, and corneal endothelial count were performed on the right eye, with the results shown in Fig. 1A, B, C, and D. Ultrasound of the eye is depicted in Fig. 2. Photo of the intraocular slit lamp of the invisible intraocular lens in Fig. 3. These examinations led to a definitive diagnosis of IOL opacification following cataract surgery in the right eye. After a thorough evaluation of the surgical risks, including the stability of the crystalline capsule bag and the potential need for vitrectomy, and after ruling out other relevant contraindications, the decision was made to proceed with IOL removal and re-implantation of a new IOL in the right eye.

The patient lay supine on the operating table. Administer 3 ml of lidocaine and ropivacaine solution for posterior orbital nerve block anesthesia, pressure was applied to stop the bleeding. Standard disinfection and draping were performed on the ocular and periorbital regions, after which a sterile adhesive film was applied. The eyelid was opened with an eyelid speculum, and the conjunctival sac was irrigated with Povidone-iodine solution for one minute, with exposure of the cornea. Then the conjunctival sac was rinsed with balanced salt solution. At 2:30, a 1-mm auxiliary incision was made on the cornea, and at 10:30, a 2.8-mm transparent corneal incision was made. An appropriate amount of viscoelastic agent was injected into the anterior chamber. We observed that the presence of the IOL causes fibrotization of the anterior capsulorhexis ring and the IOL was located within the capsular bag. A positioning hook was used to rotate and observe the crystal sac. We can observe that the crystalline capsule became hardened and it can be seen that there are changes of white fibrosis. Therefore, the surgery carries risks. There is a possibility of falling into the vitreous cavity during the operation. The positioning hook was rotated to position the cloudy intraocular lens in the anterior chamber. The artificial lens was cut open from the center using scissors, and the artificial lens was removed, with the results shown in Fig. 5. Observe that the lens capsule is stable and do not consider posterior segment surgery. Due to the hardening and fibrosis of the lens capsule, it becomes difficult to re-implant it, and there is a possibility of it falling. After cleaning the anterior chamber and the lens capsule, we observed that the lens capsule was clean and the posterior capsule was intact. Therefore, another viscoelastic agent was injected to maintain the anterior chamber, and a single A1UL22 + 20.0D artificial lens was implanted in the ciliary sulcus. The viscoelastic agent was aspirated, and both the main and side incisions were hydrated to ensure watertight closure. The procedure was completed successfully. The patient remained awake throughout the operation, with satisfactory anesthetic efficacy and no reported discomfort. Tolerance to the procedure was excellent. No intraoperative complications occurred, and the incisions remained blood-free. The patient was transferred to the ward in stable condition.

The definitive diagnosis remained IOL opacification post-cataract surgery. On the first postoperative day, the visual acuity in the right eye improved, Her best corrected visual acuity (BCVA) was 0.6 (the visual acuity was measured using the LogMAR system for clarity and consistency). Postoperative follow-up at three months and six months, the patient's best corrected visual acuity (BCVA) remained at 0.6. The postoperative patients reported significant improvement in their vision and visual quality, with no other abnormalities. Their quality of life also improved significantly. The patients sincerely thanked our efforts.

## 4 Discussion

**Fig. 4:**
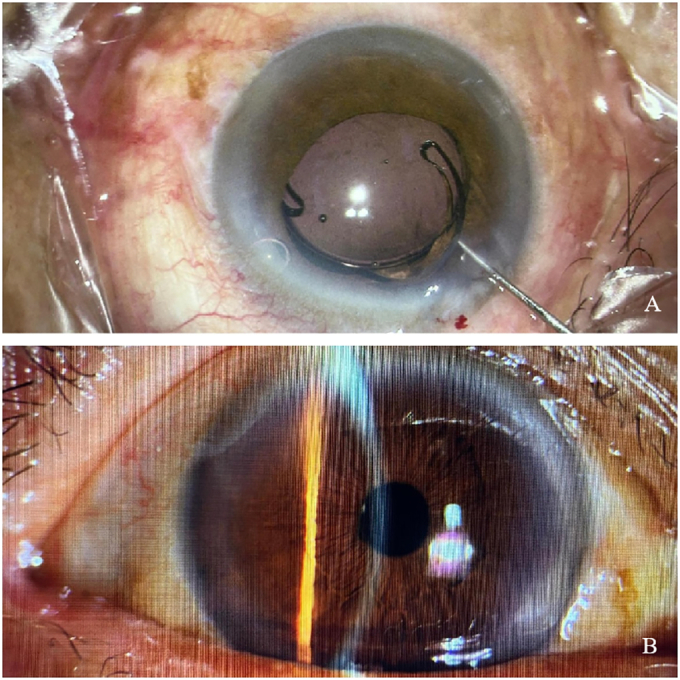
Postoperative anterior segment photography. A After a new IOL is placed intraoperatively. B Postoperative IOL translucent and correctly positioned.

Intraoperative and postoperative photographs are shown in Fig. 4A and B. At the 1-month postoperative follow-up, the visual acuity in the right eye was 0.6. IOL opacification is a relatively rare but potentially sight-threatening complication of cataract surgery. It is characterized by a reduction in the transparency of IOL after implantation. This condition must be differentiated from other postoperative complications such as posterior capsule opacification, membranous cataract, and vitreous degeneration. Posterior capsule opacification is characterized by the presence of white organic tissue and Elschnig's pearl-like vesicles of varying thickness on the posterior capsule membrane of the lens. Examination of the dilated pupil can help determine whether the shape of the opacity matches the outline of the IOL. If the opacity is located within or on the surface of the IOL, it is classified as IOL opacification. Conversely, if the opacity is confined to the posterior capsule, it is identified as posterior capsule opacification [4]. Congenital membranous cataract is a form of congenital cataract characterized by mechanical contact between the anterior and posterior lens capsule membranes. Residual lens fibers or epithelial cells may be trapped between these two layers, resulting in unevenly thick and thin opacities. Vitreous degeneration manifests as pitting, filamentous, reticular, or blocky opacities within the vitreous. In most cases, these changes do not significantly impact vision. The primary causes of IOL opacification following cataract surgery include material-related factors, surgical factors, patient-specific factors, and other miscellaneous factors. In this case, the implantation of a hydrophilic acrylic IOL 12 years ago was primarily attributed to material-related factors after excluding other potential risk factors. There are reports indicating a 77-year-old male experienced gradual vision loss in his left eye (LE) over four years post-cataract surgery a decade before. His best corrected visual acuity (BCVA) was 3/60 in the LE, with the anterior segment displaying a clear cornea but an opacified IOL within the capsular bag [5]. By comparison, there were no significant differences in the onset time and age between them. Hydrophilic acrylic IOLs are particularly susceptible to denaturation due to calcium and phosphorus deposits or UV exposure, which can lead to surface or internal opacification. The ability of hydrophilic acrylic IOLs to absorb water can contribute to several issues [6].

**Fig. 5:**
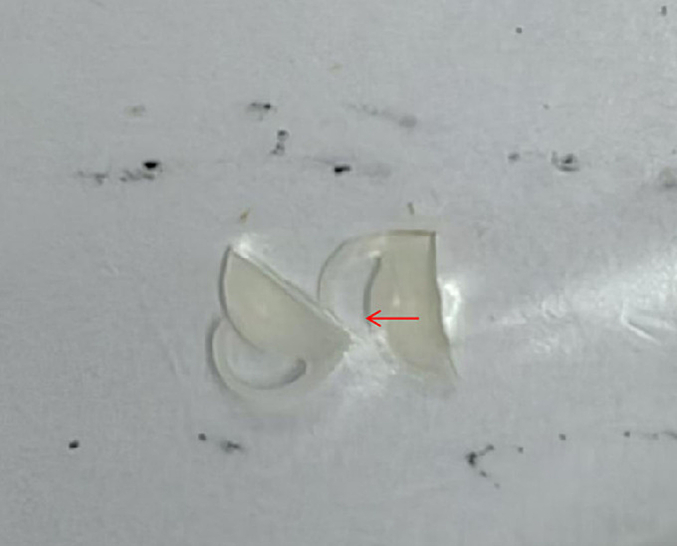
The turbid artificial lens that has been replaced. The artificial lens was cloudy and was cut.

Scale deposits: When hydrophilic materials are exposed to water over extended periods, water infiltration can cause calcium salts (hydroxyapatite) to deposit on the inside or surface of the lens. This results in “snowy degeneration” or calcified plaques, which are especially problematic in patients with diabetes and uveitis [7].

The hydrophobic acrylate IOL significantly reduces the incidence of PCO compared to hydrophilic alternatives, with reported rates of less than 6 % over three years versus over 30 % for hydrophilic lenses, attributable to its material properties and optimized edge design. It is the preferred option for long-term safety. In contrast, although the hydrophilic IOL offer favorable optical performance, they carry a considerably higher long-term risk of calcification and opacification, typically observed around 27 months post-implantation. Their use is particularly discouraged in patients with underlying metabolic disorders 410. Clinical selection of an IOL should involve comprehensive evaluation of age, systemic health conditions, and anticipated duration of implantation. According to the literature, when comparing different types of artificial lenses, the postoperative opacity rate of hydrophilic artificial lenses is higher [8].

Material swelling and structural changes: Water absorption can cause the lens to swell, altering its optical properties and potentially triggering microcracks. These changes can accelerate the opacification process [3]. Several studies have demonstrated that hydrophilic IOLs exhibit a significantly higher rate of opacification compared to hydrophobic IOLs, particularly within the 5- to 10-year postoperative period [9]. In some instances, this opacification necessitates a second surgical intervention to replace the affected IOL [10], In contrast to hydrophilic IOLs, hydrophobic acrylic IOLs demonstrate superior material stability, particularly when postoperative inflammatory responses and other factors predisposing to fibrin deposition are effectively managed [11]. When opacification of hydrophilic IOLs occurs, surgical explantation and replacement with a new lens remains the sole therapeutic option for visual rehabilitation, with clinical outcomes typically demonstrating significant visual improvement postoperatively [12]. Therefore, this case underscores the critical importance of evidence-based IOL selection in cataract surgery to mitigate vision-compromising postoperative complications. However, it should be noted that owing to specific limitations, the lack of laboratory analysis of the explanted IOL.

## 5 Conclusion

The reporting of this case not only provides important guidance for clinical practice, but also reveals the limitations of current research. Although we have identified the potential risk factors for intraocular lens opacity, more randomized controlled trials and long-term follow-up studies are still needed in clinical application to further verify these findings. While IOL opacification remains an uncommon complication of cataract surgery, its incidence has markedly declined with contemporary surgical advancements and the development of improved biomaterials. Based on current evidence, we recommend prioritizing hydrophobic acrylic or next-generation calcification-resistant IOLs in clinical practice to optimize long-term visual outcomes and reduce complication rates. By strengthening the training and education of clinicians and enhancing their understanding of this complication, the quality of patients' lives can be significantly improved, and the further development of the field of cataract surgery can be promoted.

## List of abbreviations

IOL: Intraocular lens
PMMA: Polymethylmethacrylate

## CRediT authorship contribution statement

Xiangli Wang developed the conceptual framework for the case. Qihang Lei administrated the case, edited the data, prepared the initial draft, reviewed the literature, and revised the manuscript. Qin Liu confirmed the diagnosis and analyzed the whole process.

## Informed consent

This research was deemed exempt from ethical approval in accordance with the regulations set forth by The Unit; the work has been reported in line with the SCARE criteria [13].

## Consent for publication

A written informed consent was obtained from the patient for publication of this case report.

## Ethical approval

This research was deemed exempt from ethical approval in accordance with the regulations set forth by The First Clinical Medical College of Gansu University of Chinese Medicine.

## Approval of the research protocol by an Institutional Reviewer Board

Non applicable.

## Institution of the study

Gansu Provincial Hospital.

## Guarantor

Qin Liu.

## Research registration number

Non applicable.

## Declaration of Generative AI and AI-assisted technologies in the writing process

During the preparation of this work the author used Wordvice.AI/Paraphrasing and proofreading tool in order to avoid either plagiarism or grammar errors. After using this tool/service, the author reviewed and edited the content as needed and takes full responsibility for the content of the publication.

## Funding

This work was supported by 10.13039/501100004775Natural Science Foundation of Gansu Province (No. 22JR5RA675).

## Declaration of competing interest

The authors declare no competing interest.
